# Supplementary material for: Study of Pathogenesis Using Fluorescent Strain of Cordyceps farinosa Revealed Infection of Thitarodes armoricanus Larvae via Digestive Tract
Source: Insects. 2022 Nov 10;13(11):1039. doi: 10.3390/insects13111039 (PMC9698661; doi:10.3390/insects13111039)
Supplement: Supplementary file 1 [file insects-13-01039-s001.zip › Figure S1.pdf]

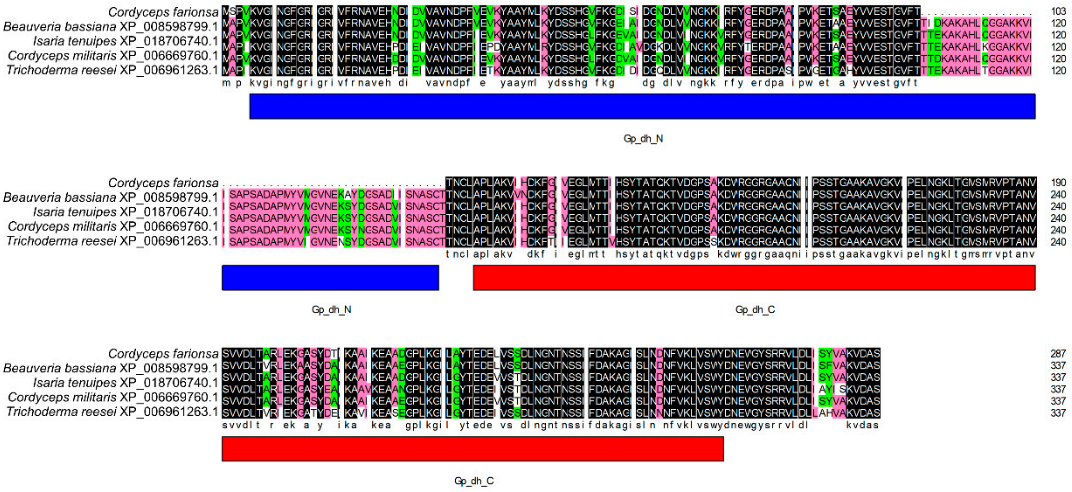

Figure S1. Homologous alignment result of GAPDH between *Cordyceps farinosa* and other fungi. The sequence with dark background is 100% homologous, the sequence with green background is ≥ 75 homologous, and the sequence with pink background is ≥ 50 homologous. The blue and red regions below the sequence represent the conservative N-terminal and C-terminal domains of GAPDH, respectively.
