# Supplementary material for: Study of Pathogenesis Using Fluorescent Strain of Cordyceps farinosa Revealed Infection of Thitarodes armoricanus Larvae via Digestive Tract
Source: Insects. 2022 Nov 10;13(11):1039. doi: 10.3390/insects13111039 (PMC9698661; doi:10.3390/insects13111039)
Supplement: Supplementary file 1 [file insects-13-01039-s001.zip › Table S1.pdf]

Table S1. Sequences of oligonucleotide primers used in this study

Name	Sequence（5′-3′）
GAPDH-F	ATGTCCCCCGTCAAGGTTGG
GAPDH-R	TCATCCTATTTGGAAGCGTCG
PgapIf-F(EcoRI)	ATGTTGGGCCCGGCGCGCCgaattcTTTTGCTGATAGCGATGCCGGATGA
PgapIf-R	CTCGCCCTTGCTCACCATGGTGTTTATTGATTAGAAAGGT
EGFP-F	ACCTTTCTAATCAATAAACAATGGTGAGCAAGGGCGAG
EGFP-R	ACGTTAAGTGGATCCCGGTTACTTGTACAGCTCGTC
TtrpC-F	GACGAGCTGTACAAGTAACCGGGATCCACTTAACGTTACTG
TtrpC-R(HindIII)	CACATTCTGCGGCCTaagcttAAGAAGGATTACCTCTAA
Bar-F	ATGAGCCCAGAACGACGC
Bar-R	CCCGTCACCGAGATCTGA
Tar-1-F	AAAGGGTCTGTGCTTGTATGGG
Tar-1-R	CGTTGACGGGAATCTGGAA

The sequences with lowercase letters, gaattc and aagctt indicate *Eco*RI and *Hin*dIII restriction sites, respectively.

The sequences with underlined letters indicate homology arm sequences.
